# “It’s a Living Experience”: Bereavement by Suicide in Later Life

**DOI:** 10.3390/ijerph19127217

**Published:** 2022-06-13

**Authors:** Trish Hafford-Letchfield, Jeffrey Hanna, Evan Grant, Lesley Ryder-Davies, Nicola Cogan, Jolie Goodman, Susan Rasmussen, Sophie Martin

**Affiliations:** 1 School of Social Work and Social Policy, Faculty of Humanities and Social Sciences, University of Strathclyde, Glasgow G1 1XQ, UK; jeffrey.hanna@qub.ac.uk (J.H.); evan@camgrant.org.uk (E.G.); lryderdavies@btinternet.com (L.R.-D.); sophie.martin.2017@uni.strath.ac.uk (S.M.); 2 School of Psychological Sciences, Faculty of Humanities and Social Sciences, University of Strathclyde, Glasgow G1 1XQ, UK; nicola.cogan@strath.ac.uk (N.C.); s.a.rasmussen@strath.ac.uk (S.R.); 3 Mental Health Foundation, London SE1 9QB, UK; jgoodman@mentalhealth.org.uk

**Keywords:** bereavement, suicide, ageing, later life, suicide prevention, moral injury, trauma, peer support

## Abstract

Bereavement by suicide for people in later life is significantly under-researched. Research on ageing and suicide has yet to address the experiences of those bereaved by suicide and how such a devastating loss affects the ageing experience. **Objectives**: We explored the substantive issues involved in bereavement by suicide and its impact on later life. **Methods**: This was a co-produced qualitative study. Peer researchers with lived experience conducted in-depth interviews with twenty-four people aged 60–92 years. A phenomenological approach informed the data analysis. **Main Findings:** Themes described included (1) moral injury and trauma; (2) the rippling effect on wider family and networks; (3) transitions and adaptations of bereaved people and how their ‘living experience’ impacted on ageing. **Conclusions**: It is important to understand how individual experiences of suicide intersect with ageing and the significance of targeted assessment and intervention for those bereaved by suicide in ageing policies and support.

## 1. Introduction

Death by suicide is a complex issue of global concern. It is estimated that 700,000 people die from suicide every year [1,2]. In the UK, 8.67% of all deaths are from suicide [3]. This has a significant impact on those bereaved by suicide (commonly defined as someone who has: “lost a significant other (or a loved one) by suicide, and whose life is changed because of the loss” (p. 43) [4]. The interpersonal impact stretches far beyond those closely related to the person [5].

The experiences of suicide bereavement of those aged >60 years old, however, remains a significantly under-researched area. A systematic review of studies found that none fulfilled the inclusion criteria [6]. Hyboldt et al.’s [7] subsequent qualitative empirical study investigated the age-related factors for participants during the post-bereavement restoration process and their re-orientation to life after such a devastating loss. The lack of theoretical and methodological consistency in suicide and ageing research has also been noted [8,9]. Research findings also call for the greater engagement of care professionals [10,11,12] and consideration of the interpersonal and structural impacts of ageism [13] in order to find answers to the multi-faceted impact of bereavement by suicide. This study sought to explore the experience of being bereaved by suicide on the individual in later life, the implications for help-seeking, support needs and how bereavement by suicide interacts with ageing experiences.

## 2. Materials and Methods

This study used an in-depth interpretative phenomenological approach (IPA) [14] to explore the substantive issues for people bereaved by suicide in later life. IPA is concerned with the human lived experience, and posits that experience can be understood through exploring the meanings which people impress upon it [15,16]. The research team comprised of academic researchers from social work, psychology, a national mental health charity engaged with ageing empowerment, and two peer researchers (aged 60+ years, with lived experience). Peer research is thought to promote accessibility by grounding data collection in the experiences of those being researched [17,18] and to produce more relevant and practice-oriented knowledge [19,20].

We recruited peer researchers through our charity partner. Peer researchers were remunerated in line with UK guidance for working with experts by experience [20]. This democratisation of the research process required the team to have clear structures to support team members with different expertise and knowledge such as regular briefings and debriefings to mitigate the impact of emotive interviews and practices that more experienced researchers may take for granted [18]. This included providing formal training on qualitative interviewing and data analysis and opportunities for both verbal and written structured dialogue to facilitate mutual and reciprocal learning. The topic guide (see Table 1) was informed by a literature review and scoping of current suicide prevention strategies for potential domains and constructs [21]. While the topic guide had a strong focus on support after loss, it sought to gain broader insights into participants’ lived experiences of their loss(es) and their specific relationship to their experiences of ageing.

The framework underpinning data collection encouraged participants to recount events and the impact of the loss surrounding the person(s) they were talking about by narrating their experience into temporal order and meaning, with a particular emphasis on how this affected their later life. This flexible approach to interviewing encouraged reflective thinking and effort to explain their situation and response to it [22].

The target population were people who were aged 60 years and over, living in the UK, with experience of the loss of a significant other by suicide at least 12 months prior to engaging in the interview. Omerov, Steineck and Dyregrov et al. [23] suggested that meaning-making, recall of experiences and post-traumatic growth are better after this period. The team used purposive and opportunistic sampling to recruit participants using direct mail shot through organisations connected with older people in the UK and via social media. Following screening and informed consent, participants completed a short demographic questionnaire, which captured their individual characteristics, their relationship to the person who died, and the length of time that had passed since the suicide.

One-to-one interviews were conducted virtually on the telephone (*n* = 10) or Zoom (*n* = 14) over 3 months in 2021 coinciding with a period of COVID-19 lockdown in the UK. These were audio-recorded with informed consent and professionally transcribed verbatim. Interviews lasted between 39 and 100 min (mAvg = 58 min). Four members of the team conducted twenty-four individual interviews; and the peer researchers conducted sixteen of these interviews.

As a descriptive, cooperative study, data analysis was inductive. Two researchers read and coded every transcript and met to discuss each transcript as well as identify and reflect upon preliminary themes. These meetings were audio recorded and the transcriptions used to verify and report on the main themes. This method drew on constant comparison [24] to help uncover participants’ meanings and furthering interpretive understandings [25]. The team observed data saturation after approximately 17 interviews. In the transcripts, we looked for complex ideas, particularly age-related issues, metaphors and critical moments. We also engaged in a critical reflection of how the qualitative data were interpretated in order to capture any biases and misperceptions during the analytical process. Analyses focused on understanding the breadth of experiences and building a picture of bereavement by suicide, and what this meant for later life grounded in the participants’ own narratives.

Ethical approval was provided by the University of Strathclyde Ethnics Committee. Participation was voluntary and followed both verbal and written consent. A key ethical consideration was the impact on researchers and participants from talking about bereavement and suicide [26,27]. The protocol drew on established guidance for working with people bereaved by suicide [28]. Participants were given a structured debriefing and signposted towards support. Participants were followed up one week after the interview to remind them about self-care. Researchers kept a critical incident/reflective diary for their own personal use alongside interviewing. The team adopted guidelines on working with vicarious trauma and had regular debriefings and access to a clinical psychologist. In the spirit of cooperative enquiry, we established clear processes for the peer-research members of the team to ensure that their contributions were valued and provided opportunities for their challenge and leadership [29].

A project advisory group comprised members working in suicide prevention, bereavement support, mental health social work, and a lay older person. This group reviewed the research protocol and tools, and commented on the findings from the interim report.

## 3. Results

Table 2 provides details on the characteristics of the participant sample (*n* = 24). The participants were predominantly female. Participants were aged 60–94 years old (mAvg = 72.0 years). Their relationship to the person who died by suicide included; being their parent (*n* = 15), spouse/partner (*n* = 4), parent in-law (*n* = 1), grandparent (*n* = 1), aunt/uncle (*n* = 1), and sibling (*n* = 2). The time elapsed between the death was between 1 and 20 (mAvg = 6.8 years).

Table 3 provides a schematic diagram and description of the three themes and subthemes discussed in this paper. Age-related experiences were integrated across three themes comprising: (1) moral injury; (2) the rippling effect on relationships; and (3) transition and adaptation through a living experience in later life.

### 3.1. Moral Injury and Trauma

Most participants provided vivid and visceral descriptions of learning about the suicide and its immediate aftermath. These encompassed intense physical and emotional pain with severe shock and numbness. Two people used the metaphor of ‘a bomb going off’ to convey the immediate and uncontrollable devastation experienced by such a traumatic loss.

A sense of moral injury in the aftermath of experiencing a loss by suicide incorporated an overall sense of failing to prevent, bearing witness to, or learning about the suicide and how this transgressed deeply held moral beliefs and expectations, which compounded the traumatic loss. The distressing psychological, social, behavioural aftermath following the suicide in turn gave rise to moral injury. Negative reactions from people in the bereaved person’s network left them feeling isolated and removed from crucial sources of support:

*“* *Because it was suicide, I could instantly tell by the person’s reaction whether to go on with the conversation. A lot of them, kind of, just back away and wouldn’t even respond. I remember walking down the town that I used to live in, this lady that I knew really well was walking towards me, she crossed over when she saw me. I’ll never forget that.”*

(female, 69, parent).

This literal crossing of the street to avoid the bereaved person came up regularly. Moral injury stemmed from the lack of insight and discomfort of other people which one participant described as ‘sheer callousness’. Self-stigma, guilt, shame and self-blame inevitably gave rise to feelings that they should have been able to prevent this, to have foreseen it, been a better parent or partner, and awareness of the manifest negative judgement and stigma from others:

*“… it’s the guilt that this person… could do something so awful to themselves. To be so desperate that they’d take their own life. You know,… I think, the guilt just gets to you, just… and you feel that everybody’s sort of, not judging you, but they must think, gosh, you know, what’s happened in this family, that this person could do this to themselves? Why did he not come to you and talk to you?”*

(female, 64 years, parent).

Despite some knowing that suicide was a risk and that it could happen, it was still a tremendous shock that made people feel responsible or that they had failed in some way:

*“… I felt I should have been able to do something to have spotted what was going on… done something to change the course of events.”*

(male, 72 years, partner).

Moral injury also occurred where participants were dismissed where they had asked for professional help. They described missed opportunities during the immediate period before the suicide:

*“he’d been threatening with suicide and that day they had the mental health team out, a crisis team and he told them he was wanting to die and they dismissed it, basically and left him in the community. They had convinced me and my daughter that, oh, we were overreacting… it was only a few hours later he killed himself.”*

(female, 62 years, grandparent).

#### 3.1.1. The Rippling Effect on Relationships and Wider Social Networks

*“I can honestly say it was the most devastating thing that’s ever, ever happened to me. I had been bereaved, I’d lost both my parents, I’d lost my friends, I had a miscarriage, I’ve been divorced. I had gone through major life affecting events but honestly this was… I just can’t tell just how devastating it was.”*

(female, 72 years, parent).

Participants not only gave testament to the devastating effect on their own lives but this touched significant others (partner, children, friends, wider family) and social networks (acquaintances, work colleagues, neighbours, community) both in the immediate aftermath and longer-term impact. From their later life perspective, some framed these impacts in the context of historical traumatic events and problematic relationships. These affected their coping mechanisms and particularly the family.

The bereaved person’s caring role often took precedence over their own immediate needs. One man talked about his mother who deteriorated quickly after the suicide with cognitive decline and died soon after:

*“she was diagnosed with Alzheimer’s in February and I truly believe, it was bought on by the shock of my brother’s death.…she went down so quickly, it was shocking”*

(female, 62 years, sibling).

Earlier traumatic events involving domestic violence, alcohol issues and the ambivalence from particular family members involved the bereaved person moderating challenging relationships. They were often responsible for all the practical arrangements. Roles became complex where there were blended or reconstituted families. Several participants bereaved as parents, articulated challenges associated with a difficult relationship with an ex-partner or the other parent. They felt unable to comfort each other, remained in dispute about the arrangements for their loved ones such as who could attend the funeral. These experiences contributed to the participants’ own lack of self-care and, for some, to extreme post-traumatic stress. Combined with blame and shame, post-suicide family interactions became extremely stressful. Such fractured relationships compounded the person’s own loss, and for some, led to a deep sense of betrayal. This extended to their wider friendships:

*“There was one friend actually who I thought was a good friend and she sent flowers and sent a card and then I didn’t hear anything from her at all for nine months… I was absolutely furious, I thought she has not been in touch with me since he died, not once to say are you okay, is there anything I can do, do you want to talk?… and then nine months later when it’s my birthday she just gets in touch and asked me if I wanted to go out for lunch. No mention of [name], no mention of how are you feeling… And I just said I don’t want you to contact me ever again …… she didn’t even come to the funeral, she didn’t ask when it was, she didn’t come, and I was so livid that after 20 years I just cut off all connection with her.”*

(female, 62 years, grandparent).

These silences were as injurious as insensitive comments. Participants spoke of awkward silences or countered expectations that they ‘*should have got over it by now*’ or *‘it was God’s will”* (Participant 22, female, 63 years, partner). They longed to be asked how they were coping, to have the person’s name spoken and experience other meaningful exchanges about the things most important to them.

Sources of practical support were, however, highly valued:

*“The practical things my daughter is absolutely amazing. She took over everything. I mean, I’m absolute… I’m at the stage in life, I’m nearly 70, I just couldn’t deal with anything anyway, but she is so good …she thinks of everything, actually. And she still does.”*

(female, 62 years, grandparent).

From a later life perspective, participants often reflected an appreciative and empathetic understanding of the impact of the suicide on the family. Losing a child in later life conflicted with the expected ‘natural order’. Their social standing or position within the family subsequently changed with new responsibilities and dependencies; taking care of grandchildren, siblings and younger family members. They found themselves compensating for the absence of the deceased person, being tentative about their own grief and holding back:

*“She needs me …… and what I did was, I became the… I, kind of, absorbed everyone’s pain. My mum, my niece, my nephew, my sister, so I didn’t actually have time to process me. And my aunt actually rang me and said, [name] what about you?”*

(female, 62 years, sibling).

Another participant (male, 78 years, partner) was concerned about their daughter, who at the time of losing her mother by suicide, had four girls all under four including twins of three months. Another commented:

*“So, the first few weeks really was me being there for them and cooking and cleaning and doing all the things that she couldn’t do. I had to be there and I had to be strong and look after my granddaughter because her mother wasn’t capable of doing it at the time, she was in such grief that she couldn’t do anything. My daughter couldn’t go to work, she was in very, very, deep depression… ”*

(female, 68, grandparent).

These intense caring responsibilities sandwiched between ageing parents and their own children, found participants caught in the firing line. For example, one 78-year-old experienced abusive phone calls after the funeral from her daughter-in-law’s parents, blaming her for the suicide. Another 69-year-old, after losing her father, described being at the mercy of her ‘controlling’ mother and being at the receiving end of her anger, which was very hurtful.

#### 3.1.2. Transition and Adaptation to Ageing through a Living Experience

The third theme reflected poignant and significant experiences in the personal journey of the bereaved person in later life. This included reflections about their own future, motivation, mortality, and accounts of help seeking. Some described periods of transformation often coinciding with activism and leadership with their peers through shared lived experience. While participants were bereaved at different age-points in their life, they reflected on what their experience meant in terms of ageing:

*“… I’m 74 and she was my only child… she was the future and how things are going to be is something very, very important at the moment. I’m giving sort of a lot of thought to it and it’s causing me a lot of sadness. So, it’s something that, yeah that I need to really… that I’ve been thinking of. At one point years ago, in terms of a Will, in terms of what I do with my property and my precious possessions and things, is something that is huge”*

(female, 74 years, parent).

This perception of having no-one to look out for you in later life was evident:

*“I think that’s the older a person is, when the bereavement happens, the more age does have an impact from isolation point of view, or lack of grand-children, or lack of somebody coming in to do your washing for you, or whatever it might be. The older you are, the less time you’ve got to sort of get your life back together again in some way or other”*

(female, 73 years, parent).

One woman living alone (aged 74) was now using psychotherapy. She reflected on the COVID-19 national lockdown and how she had ‘always been locked down’ since her daughter’s death. This was an important time for reparation and she did a ‘lot of (positive) sorting and thinking’ about her daughter. She observed the potential for a greater understanding of the impact of death following the huge loss of people during the pandemic as positive. She also used a telephone befriender service which she found helpful and comforting and described herself as “*emerging again like a metamorphosis, like a butterfly, but it’s a long, long process”.*

Two others commented on the relationship between their grief and the physical and psychological effects of growing older:

*“Well, the first one is, I find sometimes that something’s bothering me or upsetting me, or I’m feeling down, I think well is this [Name] or is just getting old?… am I attributing all of this to the bereavement, when in actual fact, I’d be feeling like this anyway?… I don’t think you can rush grief. But, at the same time, I’m conscious of the fact that if I don’t try and at least twin track, I’m not going to finish grieving before I die.”*

(female, 73 years, parent).

These health concerns triggered greater awareness of the participant’s vulnerability and potential dependencies:

*“I had a foot operation, nothing serious, about a year and a half ago and that made me really realise that there’s not going to be [name] around to come and be my next of kin. It really struck home that sort of feeling that as you approach old age and all the rest of it, you’re not going to have your nearest and dearest around. It’s something that I’ve got to manage very much on my own.”*

(female, 74 years, parent).

Another man’s insight was philosophical:

*“We struggle with that somebody has gone, and they’re actually gone forever. And again, I put it down in part, to a sort of an arrogance that we feel we’re impregnable and immortal, yet we know we’re not, and I know now, that as I age more and more”*

(Participant 15, male, 72 years. partner).

Participants reflected on the timing of the suicide within their own life course, how they learned to cope and adapt to the loss and its association with other (sometimes cumulative) losses. One woman was ‘glad’ this happened when she was older, as she had more time with her daughter and in anticipation of living a short while longer would have less pain to endure:

*“Well, I sometimes feel that I’ve not got the energy for it anymore.… I feel as if one leg of the table’s missing, if you know what I mean. That sounds a bit weird, but there’s still a limp if you know what I mean. That’s what I’m trying to say, I think. I feel at times kind of broken, yes.”*

(female, 78 years, parent).

For those retired, they talked about feeling or being very alone, their lack of plans and described temporal and visceral moments that captured these feelings:

*“You feel it, and you’ll recognise this I’m sure, [name] yourself. It’s mornings and evenings, those early hours, those moments when you put yourself into bed, and there is this, almost deafening silence”.*

(male, 72 years, partner).

Some bereaved parents remised about lost opportunities for grandchildren. This meant loss of practical support and angst about their future:

*“It feels as if my chest is being torn up”.*

(female, 63 years, parent).

Participants echoed the importance of disclosure, talking, listening and the validation of experiences following loss through suicide. They described different experiences of seeking help, the type of help offered and taken forward. Some appeared to require initial support that focused on the trauma of the suicide, whilst some accessed therapeutic/counselling services earlier on. They perceived these latter interventions as being more valuable once a significant period had passed. The timing of help seeking and help giving was arbitrary but very important. Those who developed extreme post-traumatic stress were able to connect with appropriate services, whereas many had hit or miss experiences with access and signposting to services:

*“She gave me permission to fall. And I fell hard. I became scared of everything, I didn’t like the dark, I became paranoid, I thought people were talking about me. It was the most frightening time of my life. There was no energy, I just slept all day and all night. So, one or two weeks since… I had to go back to the doctor to renew my prescription and he said, what can I do to help, would you like counselling? And I said, yes please. So, that was my turning point. But those few weeks were the darkest period of my life… I’m really lucky that I was able to meet with this counsellor, who was my saviour.”*

(female, 74 years, parent).

Help-seeking was connected to motivation to survive, or having to carry on for others:

*“You’ve got a very simply choice, you either carry on or you don’t. And I did have moments, I think they were, what I would call, poetic moments… I remember going through a period, I can’t remember quite when it was, when [name] had gone, and realising that both my daughters are married, they’ve both had two children, my mother was still alive, she only died, it’s coming up to two years now.. and I remember feeling, nobody needs me anymore. Nobody needs me. My kids don’t really need me. They’ve got their own lives, I’m potentially a complication.”*

(male, 72 years, partner).

There were references to suicidal thoughts and/or wish for the hastening of the end of life that came and went:

*“I sometimes feel I can drive my car into a wall. I just get fed up with it, I can’t take anymore. Aye, I think of suicide a lot. I don’t think I’d do it, but I sometimes wish I wasn’t here”.*

(female, 68 years, parent).

One woman whose daughter died 12 years ago reflected on how older people usually hope for a longer life. As someone who was ’60 something’ at the time, she was comforted by the thought that she would not have to suffer for too long (female, 78 years, parent). Others directly talked about their own mortality and not having enough time to get their life back together. They anticipated a loss of control related to getting older:

*“I think what I worry about is that, that when I do get older, and I get, I mean, I kind of, if I do realise, if I get to that chance to realise my mortality, and you know, I’m reaching the end of my existence. That I’ll start to become more mentally challenged… Because my belief is that if you suppress it, it doesn’t go away, it just goes deep, and when you get older, it then begins to come out, in all sorts of ways”*

(female, 73 years, partner).

Learning to adapt and live with grief was often articulated as being a mind-set adopted to radically accept the loss and continue living, despite the adverse challenges, barriers and reminders. Adapting to bereavement in different ways meant neither looking for closure but accepting that this was a possibility. One emphasised that it was a living experience:

*“I couldn’t put people through what we’ve all been through basically. Although there have been times and certainly even in lockdown last year, although I do feel most of the time I feel incredibly together, but things… you do think oh this is a battle. Which is why I think it is a living experience, I don’t think it’s a lived experience”.*

(female, 73 years old, parent).

There was some evidence of ageism as one person commented on the research:

*“I’m really pleased that this is… you know, somebody’s doing things that are about suicide, especially at our age, because when you get older, you’re not important, in general”.*

(female, 68 years, parent).

Our participants expressed a strong orientation to the importance of the shared lived experiences with others who had lost a loved one through suicide and learning to adapt to such a significant loss. These comprised of transformational moments through helping others. They described secondary gain from this sense of agency and control in one’s own life, through interactions with their peers, actively seeking out others with similar experiences and recognising the wider meaning of their own lives within the context of societal responses to suicide. Key organisations were named as hosting and enabling networking and peer support:

*“I have to say it’s been a real privilege to meet some of the people who’ve lost somebody and to realise how… and everybody does it their own way and it’s just… but it is unbelievably painful. I wish I wasn’t doing any of this because I wish I didn’t know about it, but in some way it’s given me some meaning in life. You’re not alone. They’re not alone. It’s not just feeling… we know what it’s like”.*

(female, 73 years, parent).

One participant took direct action by campaigning locally and setting up an information point at the station where his son died. Such instances of activism and raising public awareness enabled appropriate support based on direct experience being provided at both the prevention and postvention levels. One with twenty years of experience in the peer support movement expressed matured versions of herself as a compassionate and wise older woman:

*“I was around in London when there was AIDS, and we belonged to a group way back then, and I said, if there’s anything I can do? And in fact, I’ve sort of become, I don’t know what I am really… I think I’ll step down. And they keep saying, no, no. And I think I’m just a wise old woman”.*

(female, 73 years, parent).

Another talked about becoming a mental health first aider, which had transformed her life in a positive way. Other turning points were exemplified in finding new relationships and love such as one man had done at the age of 72 yrs.

## 4. Discussion

This study of the experiences of being bereaved by suicide and how it impacted the bereaved person’s later life gave rise to three rich themes from the data collected. The emotional manifestation of moral injury and trauma, negative feelings and cognitions present in those living with the traumatic loss and the wider impact on family and other social networks were emphasised in the narratives of adults in later life. Learning to live with the loss and to navigate multiple transitions and adaptations was central to how people navigated and made sense of their experiences in later life. Given the dearth of research to date, the findings offer a novel contribution to our understanding of the experiences of this population who are less recognised or visible.

Fiegleman, Gordon and Jordan [30] noted the complexity of stigmatisation that older people might face in these circumstances. Internalised ageism and factors associated with ageism, for example, insensitive comments from professionals and other people in their networks were reported by our participants. These manifested at different levels and prevented individuals from accessing support or feeling entitled to ask for support or in prioritising their own health and wellbeing. Participants did not always recognise differences in how the bereavement impacted their physical and mental health and instead attributed these to the natural consequence of ageing. Furthermore, professionals tended not to ask directly about their needs or offer psychosocial support such as counselling, perhaps attributing the persons’ problems to ageing. Bereaved parents reported particular strained and harmful consequences of their loss. A lack of recognition of the impacts within their own close networks was complicated where there were blended families or a history of family trauma. Reed [31] found more grief-struck survivors detached from their families than those who were less grief-struck. Grief therapies that use a family-focused approach include family-strengthening skills, particularly for families who show a high level of distress and find social and physical adjustment challenging [30,31]. A conceptual review of ageing and suicide [10] has demonstrated how older people experience the loss of something they had enjoyed doing or feeling, a loss of value, a feeling of tiredness and, in some cases, a feeling that they were in a process of losing themselves. All of these factors that can manifest alongside the physical aspects of caring can pose serious risk factors for self-harm and suicide in later life.

It seems important that professionals actively enquire from bereaved people in later life about the kinds of support they need, given the many examples of people in this study who felt unable to assert their needs or where these were subsumed into a caring role.

The role of the family doctor was commented on by some participants. Foggin et al.’s [32] study of general practitioners (GPs) dealing with parents who were bereaved by suicide, however, revealed an unpreparedness and uncertainty with regard to dealing with suicide and its effects on survivors and recommended that GPs were routinely informed of death by suicide to prepare for such ongoing encounters. In our study, a range of concerns were raised about the lack of skills and confidence that different professionals have in working with suicide and suicide bereavement and linked to the need for a more sensitive and timely approach to people and simply knowing what to offer.

There is much to learn from people bereaved by suicide on what support is needed and when and how to act more helpfully. In light of the intense caring roles that older people had demanded of them to step into, together with the negative feelings and cognition arising from moral injury and trauma, they may need help to navigate and evaluate which relationships they should preserve or even temporarily avoid or discontinue. Feigelman, Gordon and Jordan [30] suggested that professionals could guide bereaved people on how to ‘teach’ people close to them to overcome their own fears and lack of familiarity with suicide loss. Survivors had to accept that some people close to them or in their social networks had limited capacity to comfort or respond appropriately. Furthermore, interventions that directly involve the person’s family or social network such as partners, family and friends may have the potential to ameliorate some of the distress documented in this research. Ongoing attention to suicide awareness that challenges and reduces stigma must continue. It is clear that we need more skilled professionals in this area of work.

There was a paradox in that many bereaved survivors took up a leadership role in guiding their own support network and this promoted being able to live with their experiences at least. A novel finding lies in some of the positives that emerged for individuals despite the devastating impact of loss by suicide. There were accounts of transformative experiences (with which come opportunities such as understanding, awareness, peer support, and charitable work) that were so valuable to others and helped with the ongoing living experience and radical acceptance [33]. Many were driven to participate in communities with their peers, found strong connections and new belongings to a group. These are important anchors for coping with later-life challenges [7]. Groos and Shakespeare-Finch’s [33] evaluation of peer support groups for suicide bereavement found that an effortful thought process and the level of pain experienced were temporal and dependent on the individual’s own post-trauma trajectory or meaning-making process. Kasahara et al. [34] found differences between younger and older bereaved individuals. Older people with more significant life events found comfort through open dialogue, while younger individuals tended to conceal their emotions and suffering. Understanding these different experiences is a first step towards developing nuances in responding in later life. Barlow and Coleman’s [35] evaluation of a peer support program developed for survivors of suicide suggested that an intervention protocol that is collaboratively developed and delivered by peer supporters *and* professionals can offer cost-effective person-centred support.

While our participants had much in common with people who are bereaved by suicide, there may be generational differences in coping styles, with older people employing more stoic and avoidant coping styles in dealing with traumatic events. For some, their chronological age intersected with their expectations of themselves and others. Some of our participants gave up work earlier than they might have done. They also took up active roles and enjoyed being in groups or company where they could be more anonymous.

These are speculative explanations, suggesting that future research could systematically investigate the general population to see how views of suicide and suicide stigmatisation) may be shifting across generational cohorts. The intensification of time pressure in later life reflected by participants had both negative and positive impacts on how the process of living beyond the bereavement played out and heightened suicide thoughts in some. Some studies [36] have supported specific associations between suicide bereavement and suicide-related outcomes, justifying the inclusion of people bereaved by suicide in national suicide prevention strategies. Most of this research has examined this in relation to younger people [36]. Participants in our study demonstrated a greater awareness of their own end of life, associated with later life and possibly with a reduced fear of death or wish to die made more explicit in relation to their loss and potential losses. A scoping review of suicide and suicide-related concepts in older people [21] included a range of “grey area” behaviours (for lack of a better term) that are either less common, or present differently than they are in younger population groups. These include terms and behaviours such as “completed life”, “hastening of death” and “self-chosen death” [37,38]. The expression of these grey area behaviours may also be mistakenly viewed as a ‘normal’ part of ageing [39], creating further complexity to their identification and subsequent intervention.

The clinical implications of these findings are that those assessing older people thought to be at risk of suicide should inquire about a history of suicide bereavement and its impact on functioning and mental health [36]. Suicide bereavement is an indicator that stigma might be a marker for motivational moderators of suicidality after such a traumatic and often negative life event such as reluctance to seek help, thwarted belongingness or perceived burdensomeness [40]. The findings from this study highlight other indicators for help-seeking, where there is excessive burdens in caring and self-neglect accompanied by perceived stigma, which warrant further inquiry and inform the development of interventions that address these different impacts of traumatic losses and specifically to mitigate any risk of suicide and to directly ask about this.

There may be a need to unify research from different disciplines, with policy themes on ageing such as healthy ageing, concepts of promoting person-centredness within service provision and giving greater emphasis to participation which enables peer support. Being able to understand the individual experiences and pathways within suicide research can help inform and enrich assessment and evidence-based interventions [21].

Finally, the design of this study enabled the data collection and data analysis to be enriched by the direct insights and contributions of those peer-researchers with lived experience. These methods can be resource intensive, given the time and pace needed to ensure authenticity in this approach, some of which did not always coincide with the need to make progress in terms of project milestones. There was one occasion where the research team took time out and utilised the skills of one team member, a digital artist, in a session to reflect and visualise imagery emerging from the data. This was a valuable team experience enabling restoration and self-care, something not always prioritised in the academy. (One of the images has been included with this paper’s abstract). Furthermore, feedback from research participants and our peer researchers reminded the team to take a strengths approach, which offers a perspective on hope and respect for people with lived experience and this was just as important as our robust procedures on signposting and debriefing required in the ethical approval process.

## 5. Conclusions

The qualitative and exploratory approach provided rich and salient transferable insights concerning bereavement by suicide in later life from the perspectives of those with lived experience. This undoubtedly helps inform future suicide awareness and prevention research. The findings confirmed existing evidence on the experience of bereavement by suicide such as the persistence of stigma, shame and moral injury associated with suicide. We assert the importance of suicide prevention policies in ageing support and the need to target services better. There is a strong indication for the inclusion of older people bereaved by suicide in national suicide prevention strategies and focusing efforts to reduce harm and the adverse impact on ageing experiences. In later life, bereavement by suicide must be considered alongside the effects of other loss experience(s) and in the context of the interpersonal and structural impacts of ageism in society [13]. These are potential areas for marginalising older people from mainstream support. Identifying these individuals may constitute an interdisciplinary approach and improved surveillance including routine assessment in a range of services that interact with people in later life. Professionals in ageing services could also develop competencies around the assessment of the impact of suicide exposure and interventions, particularly given that bereaved people are at increased risk of suicide themselves. Professionals need the right language, confidence, and skills to discuss issues of suicide with people in later life and to articulate concerns where issues are observed [10,11,12].

Suicide, personal meaning, trauma and membership of marginalised communities should be elevated in the public health conversation surrounding ageing. Critiques of ‘successful ageing’ encourage us to recognise the intersection of individual and social factors and to consider the life course perspective in research, policy and practice [41]. We recommend further studies comparing the bereavement experiences of those bereaved by suicide in later life with other traumatic bereavements and losses using participatory mixed methods and longitudinal approaches [42]. Framing these within the context of ageing [43] may involve unifying research from different disciplines, with policy themes on ageing such as healthy ageing, personalised support and being able to understand the individual experiences and pathways within suicide research to help inform and enrich assessments and interventions in ageing care.

## 6. Strengths and Limitations

This study explored experiences of suicide in a hard-to-reach population. The co-produced nature of this study allowed stakeholders and participants to feel comfortable to share experiences which enriched the data and thus improved the quality of the study. More specifically, we believe that employing a co-produced design enhanced communication during the interviews, which adds to the transferability of the materials. However, the sample was self-selective and attracted some participants active in peer support. The findings may therefore only be partially representative. In addition, there were few men or engagement with people from diverse minority backgrounds in this study.

## Figures and Tables

**Table 1:** Areas covered in the Interview Topic Guide.

- Individual’s experience of support after their loss at different time points. - Their reflections on who or what supported them and how helpful it was; their own help-seeking behaviour and self-identified needs; What was unhelpful? - What worked best in managing their lives after the loss? How they feel they experienced their bereavement, any particular crises or transformational moments; the role of family, friends; the role of any services; the pros and cons of being supported by people who knew you and the person you lost and those who didn’t know you both. - What support would they recommend to others? Any tips for people who have are bereaved by suicide; practical things they did to manage grief; advice to family and friends that would help them supporting others; what would they like to tell professionals to do and not do. - How the experiences have affected their later life and experience of ageing?

**Table 2:** Characteristics of the 24 participants included in the study.

Variables	*n*	Variables	*n*
**Gender of participant**		**Ethnicity of participant**	
Female	21	Black, African	1
Male	3	White, British	1
**Age of participant**		White, English	13
60–64 years old	6	White, European	2
65–69 years old	4	White, Northern Irish	1
70–74 years old	7	White, Scottish	5
75–79 years old	3	White, Welsh	1
80–84 years old	2	**Disability**	
85–89 years old	1	Yes	3
90–94 years old	1	No	21
**Relationship to the deceased**		**Religion/belief of participant**	
Aunt/uncle	1	Buddhism	1
Grandparent	1	Christianity	11
Parent	15	Judaism	1
Parent in-law	1	No religion	9
Sibling	2	Prefer not to say	1
Spouse/partner	4	Quaker	1
**Sexual identity of participant**		**Location of participant**	
Bisexual	2	England	17
Heterosexual	22	Northern Ireland	1
		Wales	5
		Scotland	1

**Table 3:** Schematic diagram of key themes from the qualitative data.

Theme	Description of Theme	Sub-Themes
**Moral Injury and Trauma**	The overall sense of failing responses and trauma to prevent, bearing witness to, or learning about the suicide and how this transgresses deeply held moral beliefs and expectations linked to the sense of negative judgement and stigma from others. Moral injury was strongly associated with feelings of guilt and shame associated with traumatic loss.	Poor engagement and lack of appropriate care from professionals
		Critical of own failure to prevent/save
		Feeling helpless and calls for help unrecognised
		Being left alone to deal with grief
		Lack of insight from others including avoidance
		Being given unsolicited advice/careless thoughtless comments from others
		Lack of physical/practical support
		Perception of having failed from others/shame/stigma and guilt
		Grappling with conflicted feelings towards the person who died
		Use of metaphors to express dramatic experiences and incongruities in situations that emerged
**Rippling effects**	Positive and negative The effect on self (beliefs about self), significant others (partners, children, friends, wider family) and wider social networks (acquaintances, work colleagues, neighbours, community) is evident both in the aftermath as well as the longer term impact and consequences.	Igniting of existing or previous traumas
		Expositing quality of relationships
		Lack of care from people close to them
		Taking up care roles and new responsibilities
		Invisibility as a mature person
		Own unmet needs/disappointment
		Providing substitute care
		Being unable to assert own needs
		Impact on physical and mental health of own and others
		Fear/awareness of suicide in self/others
		Significance of key people reaching out
		Making sense of disruption to expected natural order
**Adaptation and transformation to the living experience in later life**	How the bereaved person reflected with time on the impact of suicide on themselves and their lived experiences particularly as they became older. How they learned to adapt following the loss by suicide and connected with peers. This related to the importance of disclosure talking, listening and validation of experiences following loss through suicide and meaning-making about their own lives and life with the person.	Timing of help seeking
		Quality of responses to help seeking e.g., family doctor
		Recognising different types of pain
		Suicidal thoughts and behaviour
		Radical acceptance of loss
		Temporal perspectives/time lost vs. time left
		Marking anniversaries/meaning of significant events
		Peer support/peer education/activism
		Role of professionals in recognising bereavement by suicide

## Data Availability

Information about the data reporting results can be obtained by contacting Professor T.H.-L.
